# The Vicious Cycle of Melanoma-Microglia Crosstalk: Inter-Melanoma Variations in the Brain-Metastasis-Promoting IL-6/JAK/STAT3 Signaling Pathway

**DOI:** 10.3390/cells12111513

**Published:** 2023-05-30

**Authors:** Sivan Izraely, Shlomit Ben-Menachem, Sapir Malka, Orit Sagi-Assif, Matias A. Bustos, Orit Adir, Tsipi Meshel, Maharrish Chelladurai, Suyeon Ryu, Romela I. Ramos, Metsada Pasmanik-Chor, Dave S. B. Hoon, Isaac P. Witz

**Affiliations:** 1 The Shmunis School of Biomedicine and Cancer Research, The George S. Wise Faculty of Life Science, Tel Aviv University, Tel Aviv 6997801, Israel; sivanisr@tauex.tau.ac.il (S.I.);; 2 Department of Translational Molecular Medicine, Saint John’s Cancer Institute, Providence Saint John’s Health Center, Santa Monica, CA 90404, USA; 3 Department of Genome Sequencing, Saint John’s Cancer Institute, Providence Saint John’s Health Center, Santa Monica, CA 90404, USA; 4 Bioinformatics Unit, The George S. Wise Faculty of Life Science, Tel Aviv University, Tel Aviv 6997801, Israel

**Keywords:** melanoma, microglia, brain metastasis, inter-tumor heterogeneity, IL-6, STAT3, SOCS3

## Abstract

Previous studies from our lab demonstrated that the crosstalk between brain-metastasizing melanoma cells and microglia, the macrophage-like cells of the central nervous system, fuels progression to metastasis. In the present study, an in-depth investigation of melanoma-microglia interactions elucidated a pro-metastatic molecular mechanism that drives a vicious melanoma-brain-metastasis cycle. We employed RNA-Sequencing, HTG miRNA whole transcriptome assay, and reverse phase protein arrays (RPPA) to analyze the impact of melanoma-microglia interactions on sustainability and progression of four different human brain-metastasizing melanoma cell lines. Microglia cells exposed to melanoma-derived IL-6 exhibited upregulated levels of STAT3 phosphorylation and SOCS3 expression, which, in turn, promoted melanoma cell viability and metastatic potential. IL-6/STAT3 pathway inhibitors diminished the pro-metastatic functions of microglia and reduced melanoma progression. SOCS3 overexpression in microglia cells evoked microglial support in melanoma brain metastasis by increasing melanoma cell migration and proliferation. Different melanomas exhibited heterogeneity in their microglia-activating capacity as well as in their response to microglia-derived signals. In spite of this reality and based on the results of the present study, we concluded that the activation of the IL-6/STAT3/SOCS3 pathway in microglia is a major mechanism by which reciprocal melanoma-microglia signaling engineers the interacting microglia to reinforce the progression of melanoma brain metastasis. This mechanism may operate differently in different melanomas.

## 1. Introduction

Patients with melanoma as well as with several other cancer types are prone to develop brain metastasis and the prognosis for such patients remains poor [1]. In addition to genetic factors [2], brain metastasis formation is governed by interactions of brain-metastasizing tumor cells with cellular components of the brain microenvironment [3,4]. All brain cell types, such as nerve cells, endothelial cells, astrocytes, and microglia, can and indeed do interact with brain-invading cancer cells. Such interactions may promote or, alternatively, restrain brain metastasis [4,5]. Our lab focused on the crosstalk between melanoma and brain cells including endothelial cells, astrocytes, and microglia [6,7,8,9,10,11,12,13,14]. In this study, we set out to identify targetable molecular pathways in the melanoma-microglia interactome that either promote or inhibit progression to melanoma brain metastasis (MBM).

Microglia, the macrophage-like resident cells of the brain, perform important functions in the development, homeostasis, and pathology of the brain [15]. Microglia as distinct from most myeloid cells arise from the embryonic yolk sac rather than from hematogenous organs [16]. These cells maintain their distinct character by self-renewal [17]. As the major immune cells of the central nervous system, microglia cells perform important functions in neuroinflammation triggered by infection or injury. In the intact brain, microglia constantly change their morphology by extending and retracting their cellular extensions, to actively monitor the brain parenchyma and respond to various insults. Responding activated microglia may exert either neuroprotective or neurotoxic effects. Microglia function also as “housekeepers”, by scavenging brain intruding microbes, foreign material, and damaged cells. Microglia are involved in monitoring the neuronal activity and integrity of synaptic functions by removing damaged or redundant synapses [18]. 

Previous studies indicated that microglia cells take part in the formation and maintenance of brain metastasis [1,19,20]. A study from our lab demonstrated that human melanoma cells and microglia reprogramed each other’s phenotype, thereby promoting melanoma progression towards brain metastasis [8]. Intra- and inter-heterogeneity [21] is a recognized reality that should be considered when drawing conclusions as to tumor cell behavior and response to signaling. Indeed, we reported previously that melanoma cell lines differ from each other in their proteome [22] and in their response to intrinsic and external signaling. Furthermore, the outcome of melanoma-microglia cross talk is likely to be determined by inter melanoma heterogeneity as well as by the heterogeneity of microglial cell populations [23].

Taking these factors into account, we set out to elucidate the molecular pathways triggered by melanoma-microglia interactions. We found that signal transducer and activator of transcription 3 (STAT3) activation is a central component in melanoma-microglia interactions, thus confirming published results in glioblastoma [24,25,26]. However, by employing multiple melanoma cell lines, we detected a heterogenous pattern of STAT3 activation in microglia cells exposed to signals from different melanoma cell lines. These heterogenous patterns of STAT3 activation constitutes the major issue of this study. 

## 2. Materials and Methods

### 2.1. Cell Culture 

Melanoma brain metastasis (MBM) variants YDFR.CB3, DP.CB2, M12.CB3, and M16.CB3 were established from the parental cell lines YDFR (kindly provided by Prof. Michael Mischa, Department of Applied and Experimental Oncology, Vienna University, Austria), DP-0574-Me, UCLA-SO-M12, and UCLA-SO-M16 (kindly provided by Dr. Dave S.B. Hoon) [7,27]. In short, human MBM cells were inoculated subdermally into nude mice to establish the cutaneous (C) variants. Cells from these cultures were inoculated intracardially and passaged in the brain for two or three passages yielding the brain metastatic variants (CB2 or CB3, respectively). Melanoma cells were maintained in RPMI-1640 medium (Biological Industries, Kibbutz Beit Haemek, Israel) supplemented with 10% fetal calf serum (FCS), 2 mmol/mL L-glutamine, 100 units/mL penicillin, 0.1 mg/mL streptomycin, and 12.5 units/mL nystatin. Immortalized human microglia-SV40 cells (ABM, Milton, ON, Canada) were maintained on 100 μg/mL collagen I, rat tail (Corning, Bedford, MA, USA) in Prigrow III medium (ABM) supplemented with 10% FCS, 2 mmol/mL L-glutamine, 100 units/mL penicillin, 0.1 mg/mL streptomycin, and 0.00025 units/mL Amphotericin B. Human embryonic kidney 293T cell line was maintained in DMEM medium supplemented with 10% FCS, 2 mmol/mL L-glutamine, 100 units/mL penicillin, 0.1 mg/mL streptomycin and 12.5 units/mL nystatin. To produce mCherry or GFP expressing cells, cells were transduced with a pQCXIP-mCherry or pQCXIP-GFP plasmid and selected using 1 μg/mL puromycin (InvivoGen, San Diego, CA, USA). A 0.5% FCS-supplemented medium was used for starvation in all the experiments. Cells were routinely cultured in an incubator with humidified air with 5% CO_2_ at 37 °C. 

In melanoma-microglia co-culture experiments, mCherry-labeled melanoma cells and GFP-labeled microglia cells were seeded at a 1:1 ratio, and supplemented with a mixture of RPMI and Prigrow mediums (1:1).

### 2.2. Preparation of Melanoma- or Microglia-Conditioned Medium

Melanoma or microglia cells were cultured for 24 h and were then starved for 24 h. Melanoma-conditioned medium (MCM) or microglia-conditioned medium (MGCM) were collected, centrifuged for 5 min at 220× *g*, and filtered (0.45 μm, Whatman GmbH, Dassel, Germany).

### 2.3. Isolation of Extracellular Vesicles (EVs) 

Melanoma cells were seeded to reach 70% confluency in T-175 flasks. After 24 h, cells were washed twice in PBS 1X and were starved with a mixture of RPMI and Prigrow mediums (1:1) containing 0.5% EV-depleted FCS. MCM was collected after 72 h and centrifuged at 2500× *g* for 30 min to eliminate dead cells and debris. The remaining supernatants were filtered (0.45 μm, Whatman GmbH). The infiltrate was then divided into two fractions: the MCM fraction and the EV-containing fraction. 

The latter fraction was further processed by subsequent centrifugation at 100,000× *g* for 72 min to pellet the EVs. The supernatant was used as the soluble factors (SF) fraction, or the EV-depleted MCM fraction, and the pellet was resuspended in PBS 1X to wash away co-precipitates and centrifuged again at 100,000× *g* for 72 min to re-pellet the EVs. EV pellet was resuspended in PBS 1X for nanoparticle tracking analysis (NanoSight NS300, Malvern Panalytical Ltd., Malvern, UK) or transmission electron microscope (TEM), in RIPA buffer for Western blotting, or in starvation medium for treatment of microglia cells prior to RT-qPCR. 

The concentration and size distribution of the particles were determined with NanoSight, and the morphology of EVs was evaluated with TEM. The size of the EVs was analyzed by nanoparticle tracking analysis (NTA) and the average size was 144.0 ± 2.4 nm, 181.9 ± 2.3 nm, 197.0 ± 2.5 nm, and 198.0 ± 0.3 nm for YDFR.CB3, DP.CB2, M12.CB3, and M16.CB3, respectively. The morphology of EVs was evaluated with transmission electron microscope (TEM) and EVs size was measured and ranged from 30 nm to 450 nm, validating the size measured with NanoSight. 

The EVs yield from 50 mL supernatant of melanoma was as follows: 34.2 µg for YDFR.CB3, 44.9 µg for DP.CB2, 45.9 µg for M12.CB3, and 57.4 µg for M16.CB3 cells, and were diluted to 20 µg/mL.

The EVs obtained were tested for EV markers CD63, CD81, and Alix (as well as calnexin and β-tubulin as a negative control). 

Microglia cells were treated with 2 mL MCM, 2 mL SFs, or 20 µg/mL EVs diluted in starvation medium for 24 h.

### 2.4. Cytokines

Microglia cells were treated with the following cytokines: IL-6 (20 ng/mL), IL-11 (10 ng/mL), MIF (10 ng/mL), PDGF-AA (20 ng/mL), OPN (100 ng/mL), and MCP-1 (100 ng/mL). All cytokines were purchased from PeproTeck (Rocky Hill, NJ, USA). Microglia cells were then processed for RT-qPCR, Western blotting, or FACS analysis.

### 2.5. Animals

Male athymic nude mice (BALB/c background) were purchased from Harlan Laboratories Limited (Jerusalem, Israel). The mice were housed and maintained in laminar flow cabinets under specific pathogen-free conditions in the animal quarters of Tel-Aviv University following regulations and standards of the Tel Aviv University Institutional Animal Care and Use Committee (ethical approval code: 04-19-027). The mice were used when they were 7–8 weeks old. 

### 2.6. Intracardiac Inoculation of Tumor Cells

To generate brain metastasis, mice were inoculated intracardially with 5 × 10^5^ melanoma cells under a small animal ultrasound (Vevo 770 High-Resolution System; VisualSonics Inc., Toronto, ON, Canada). MBM formation was followed by MRI at four- and six-weeks following inoculation, as previously described [27]. Six weeks following the inoculation, mice were perfused with 4% paraformaldehyde, sacrificed, and their brains were fixed and embedded in optimal cutting temperature compound (Tissue-Tek^®^ O.C.T., Sakura Finetek USA, Inc., Torrance, CA, USA).

### 2.7. Immunostaining

OCT-embedded 5 µm brain sections were cut. The sections were blocked for 60 min. Primary antibodies against SOCS3 and Iba-1(ionized calcium-binding adapter molecule 1) (antibody information is detailed in Table S1) were incubated for 1 h at room temperature (RT), followed by fluorescently conjugated secondary antibodies for 60 min at RT. Coverslips were mounted using DAPI Fluoromount (Southern Biotech, Birmingham, AL, USA). The images were viewed with a × 63/1.4 oil objective, using a Leica SP5 confocal microscope and Leica SP5 software (https://www.leica-microsystems.com/products/confocal-microscopes/p/leica-tcs-sp5/, accessed on 27 March 2023) (LAS-AF, Leica Microsystems, Wetzlar, Germany) or a confocal microscope (LSM 510, Carl Zeiss, Oberkochen, Germany) and LSM image browser.

### 2.8. RNA Preparation and Reverse Transcription Quantitative Real-Time PCR (RT-qPCR)

Total cellular RNA was extracted using EZ-RNA Total RNA Isolation Kit (Biological Industries). RNA concentrations were determined by the absorbance at 260 nm (A260) and quality control standards were A260/A280 = 1.8–2.0. RNA samples were used for cDNA synthesis using the qScript cDNA Synthesis Kit (Quantabio, Beverly, MA, USA) according to the manufacturer’s instructions. Amplification reactions were performed with SYBR Green I (Thermo Fisher Scientific, Bedford, MA, USA) in triplicates in a Rotor-gene 6000 TM thermal cycler (Corbett life science, Mortlake, Australia). PCR amplification was performed over 35–40 cycles (95 °C for 15 s, 59 °C for 20 sec, and 72 °C for 15 s). Primer sequences are detailed in Table S2. 

### 2.9. Western Blotting and Phosphorylation Assays

Cells were washed with ice-cold PBS 1X and lysed as previously described [7]. Proteins were separated on 4–12% Bis–Tris gels (Thermo Fisher Scientific) and transferred onto nitrocellulose membranes. The membranes were blocked at RT with 3% BSA diluted in TBS–Tween for 1 h. Primary Abs against the following proteins were used: phospho-STAT3 (Tyr705), STAT3, SOCS3, and anti-β-tubulin (Loading Control) (Table S1). Horseradish peroxidase-conjugated goat anti-mouse, or goat anti-rabbit (1:10,000, Jackson ImmunoResearch Laboratories, West Grove, PA, USA) were used as secondary Abs.

The bands were visualized by chemiluminescence ECL reactions (Merck Millipore, Darmstadt, Germany).

### 2.10. ELISA Assay

For the estimation of basal IL-6, cells were plated and grown in starvation media for 28 h. For conditioned medium (CM) treatments, cells were plated and stimulated with CM for 4 h, they were then washed and starved for 24 h. For co-culture experiments, melanoma-microglia cultures were plated and grown in starvation medium for 28 h. 

The supernatants were then collected, centrifuged, filtered, and 30- or 70-fold concentrated (to detect melanoma- or microglia-derived IL-6, respectively) at 4000× *g* using Amicon^®^ Ultra-15 centrifugal filter units (Merck Millipore) for 1 h. The fraction (MW > 3 kDa) was used to determine the extracellular levels of IL-6 by ELISA according to manufacture instructions using the Human IL-6 Mini TMB ELISA Development Kit (900-TM16, PeproTech). 

### 2.11. Isolation of Microglia Cells by Sorting

mCherry-labeled melanoma cells and GFP-labeled microglia cells were co-cultured for 24 or 48 h. Then, cells were analyzed on a FACSAria (Becton Dickinson, San Jose, CA, USA) and sorted for GFP expression, to isolate the microglial population, prior to gene or protein expression analysis.

### 2.12. Flow Cytometry

Anti-IL-6R Ab (Table S1) was used for IL-6R detection by flow cytometry as previously described [28]. Detection of myeloid M1/M2 markers [29,30] in sorted microglia following a 48 h co-culture with melanoma cells was performed similarly using Abs against CD16, CD32, CD86, CD150, CD163, and CD206 (Table S1). 

Antigen expression was determined using Flow cytometer S100EXi (Stratedigm, San Jose, CA, USA) with CellCapTure software (https://stratedigm.com/cellcapture/, accessed on 27 March 2023) (Stratedigm, Inc.) and FlowJo v10 (FlowJo, Ashland, OR, USA). Dead cells were gated out from the analysis. 

### 2.13. Confocal Microscopy

Microglia cells were cultured on collagen-coated cover glass (13 mm; Deckglaser, Freiburg, Germany) for 24 h at 37 °C and they were then treated with YDFR.CB3 CM for 4 h. Cells were fixed with 4% paraformaldehyde for 30 min on ice and permeabilized with 0.2% Triton X-100 for 3 min. A solution of 0.5% BSA and 10% normal goat serum in PBS 1X was used for blocking (30 min at RT). Antibodies against SOCS3 (Table S1), diluted in blocking solution, were applied ON at 4 °C. After washing, Rhodamine Red™-X (RRX) AffiniPure Goat Anti-Rabbit IgG (H + L) (1:100, Jackson ImmunoResearch Laboratories) and Phalloidin, Fluorescein Isothiocyanate (FITC) labeled (5 µg/mL, p5282, Sigma-Aldrich, St. Louis, MO, USA) were applied for 30 min at RT. 4′,6-diamidino-2-phenylindole dihydrochloride (DAPI) Fluoromount g (SouthernBiotech) was used for mounting. Slides were analyzed using a Leica TCS SP8 confocal microscope (Leica Microsystems).

### 2.14. Construction of the Expression Vector and Stable Overexpression of SOCS3

The overexpression construct of human SOCS3 (NM_003955) was created by PCR amplification of genomic DNA by Phusion^®^ High-Fidelity DNA Polymerase (Thermo Fisher Scientific) using the following primers (designed based on the GenBank Nucleotide Database of the NCBI website): SOCS3: S-5′-CAACAAACCGGTGCTGGCTCCGTGCGCCATGG-3′, AS-5′-GCGTATGGATCCTTAAAGCGGGGATCGTACT-3′. The generated fragment was digested with *Age*1 and *Bam*H1 and ligated into the corresponding sites of pQCXIN vector (Clontech Laboratories, Inc., Mountain View, CA, USA). PCR products of SOCS3 were sequenced and verified to be identical to the published sequence. Production of plasmid and viral vectors as well as melanoma transfection were performed as previously described [8]. 

### 2.15. RNA Sequencing Analysis

MCM prepared from four MBM cell lines or starvation medium were added to microglia cells for 24 h. RNA was extracted using miRNeasy mini kit (Qiagen, Valencia, CA, USA). Concentration of purified total RNA was measured using the Quant-iT RiboGreen RNA assay (Life Technologies, Carlsbad, CA, USA), and RNA quality was assessed by the RNA ScreenTape assay on the Agilent TapeStation 2200 (Agilent Technologies, Santa Clara, CA, USA). Using 1 µg of high quality (RIN > 7.0) total RNA, mRNA libraries were prepared with the KitKAPA mRNA HyperPrep kit (Roche Diagnostics, Basel, Switzerland). The quality and quantity of final libraries were assessed by High Sensitivity D1000 assay (Agilent Technologies) and Qubit dsDNA HS ScreenTape (Agilent Technologies), respectively, followed by KAPA Library Quant Kit (Illumina Inc., San Diego, CA, USA) and the Universal qPCR Mix according to the manufacturer recommendations. Libraries were pooled and sequenced on an Illumina NextSeq 550 (Illumina Inc.) using 76 bp paired-end reads.

Raw RNA-seq reads were checked for overall quality and filtered for adapter contamination using Trimmomatic (version 0.36) [31]. The filtered reads were then mapped to the GENCODE comprehensive gene annotation reference set (version 19) using the STAR aligner (version 2.4.2a) [32] with default parameters. Read counts for each feature were generated using the “--quantModeGeneCounts” function in STAR. Significantly differentially expressed genes (DEGs) were identified using ANOVA with a significance cutoff *p* < 0.05 and fold change (FC) FC ≤ −1.5 or FC ≥ 1. 5. Principal component analysis was performed using Partek Genomics Suite software (version 7.19.1125).

### 2.16. HTG miR WTA Assay

MCM prepared from four MBM cell lines or starvation medium were added to microglia cells for 24 h. RNA was extracted using miRNeasy mini kit (Qiagen). Lysis buffer was added to extracted RNA, and then processed on an automated HTG EdgeSeq instrument for probe-capture of validated human miRNAs (2083 miRNA) for 20 h [33]. After probe-capture, the samples were processed for NGS library preparation as previously described [33]. miRNA sequencing with Illumina platform was conducted according to HTG instructions [33]. FASTQ files were analyzed with HTG EdgeSeq Parser software version v5.1.724.4793 to generate raw counts for 2,083 miRNAs per sample. Each HTG miRNA WTA included negative (CTRL_ANT1, CTRL_ANT2, CTRL_ANT3, CTRL_ANT4, CTRL_ANT5) and positive (CTRL_miR_POS) miR controls that were included in the 2083 total miR panel. In all runs, Human Brain Total RNA (Ambion, Inc., Austin, TX, USA) was used as an in-process control. Data were provided as raw, QC raw, CPM, and median normalized [33].

The statistical analysis was performed using Partek Genomics Suite (version 7.19.1125) and differentially expressed miR-lists were obtained, with cutoff *p* < 0.05 and FC ≤ −1.25 or FC ≥ 1.25.

### 2.17. Reverse Phase Protein Analysis (RPPA)

MCM prepared from four MBM cell lines or starvation medium were added to microglia cells for 24 h. Protein lysates from microglia cells were extracted as previously described [7]. RPPA was performed by the RPPA Core Facility at the University of Texas, MD Anderson Cancer Center (G. Mills). 

### 2.18. Bioinformatic Analysis

A Venny tool was used to compare DEG, differentially expressed miRNAs or differentially expressed proteins. Enriched biological processes of the DEGs were obtained through the analysis of disease and bio-functions (threshold, z-score ≤ −2 or z-score ≥ 2) using Ingenuity Pathway Analysis (IPA, Qiagen) (https://digitalinsights.qiagen.com/products-overview/discovery-insights-portfolio/analysis-and-visualization/qiagen-ipa/, accessed on 27 March 2023).

For the preparation of DEGs-miRNA interactome, we identified mRNA targets of the differentially expressed miRNAs using MiRNet [34]. The identified targets were compared to those identified as differentially expressed in the RNA seq analysis. Pairs of miRNA-differentially expressed targets are shown using Cytoscape [35].

Networks of protein–protein interactions between differentially expressed proteins in the RPPA (FC ≤ −1.25 or FC ≥ 1.25) were generated using STRING protein–protein interaction (PPI) database [36]. 

### 2.19. IL-6/JAK/STAT3 Pathway Inhibition

αIL-6Ra blocking Ab (Table S1), JAK1/2 inhibitor Baricitinib (JAKi, 2 µM, A892931, Amadis Chemical, Hangzhou, China), and STAT3 Inhibitor V, Stattic (5 µM, 573099, Sigma-Aldrich) were used to inhibit the IL-6/JAK/STAT3 pathway in microglia cells. The inhibitors were supplemented to the cells simultaneously with MCM. Control cells were treated with mouse IgG1 isotype control (1 μg/mL, MAB002, R&D Systems, Minneapolis, MA, USA) or DMSO, in matching concentrations.

### 2.20. Migration through Extracellular Matrix 

An amount of 1 × 10^5^ melanoma cells were loaded onto collagen-coated transwell inserts (8 or 12 μm; Corning Costar Corp., New York, NY, USA) and were allowed to migrate for 24 h towards control or SOCS3 overexpressing microglia cells. Migrating cell fixation and analysis were performed as previously described [7].

### 2.21. Cell Proliferation Assay

mCherry-labeled melanoma, GFP-labeled microglia, or a mixture of labeled melanoma-microglia cells were seeded on a collagen-coated 96-well plate. After 24 h, the cells were washed twice with PBS 1X, and starvation medium alone, with rIL-6 or with STAT3 inhibitors was added. The plates were imaged every 4 h for 120 h, and images were analyzed to determine cell number using the IncuCyte system (Essen BioScience, Inc., Ann Arbor, MI, USA). 

### 2.22. Spheroid Formation Assay

A total of 1 × 10^3^ melanoma and 1 × 10^3^ microglia cells in starvation medium, with rIL-6 or with STAT3 inhibitors were added into 96-well low-attachment u-shaped plates (Greiner Bio-One, Frickenhausen, Germany) and centrifuged for 10 min at 1000 rpm. The wells were imaged every 1 h for 4–5 days, and spheroid measurements were analyzed using the IncuCyte system (Essen BioScience). 

### 2.23. Patients with MBM Diagnosis

Three patients who underwent upfront craniotomy for MBM at Providence Saint John’s Health Center Hospital, Santa Monica, in 2017 were enrolled. All the studies followed the World Medical Association Declaration of Helsinki. All human samples and clinical information for this study were obtained according to the protocol guidelines approved by Providence SJHC under SJHC/SJCI Joint Institutional Review Board (IRB) and Western IRB: MORD-RTPCR-0995. Informed consent was obtained from all participants. All specimens in the study were de-identified and HIPAA regulations were followed.

### 2.24. NanoString GeoMx DSP Analysis 

The FFPE tissue preparation was performed according to the manufacturer’s protocol (NanoString, Seattle, WA, USA). The 5 µm sectioned slides were stained with fluorescently labeled morphology markers (SYTO13, MART-1, and Iba-1) and incubated with the RNA probe mix for cancer transcriptome atlas (CTA), which covers 1812 unique cancer genes (NanoString). The details for Ab utilized for morphological markers are summarized in Table S3. A total 18 regions of interest (ROI) from MART-1 positive segments and 18 ROIs from Iba-1 positive segments were selected for transcriptomic analysis. ROIs were selectively illuminated with UV light to release the barcoding oligos. The libraries were generated for each ROI by PCR, quantified, denatured, and sequenced on a NextSeq550 sequencer (Illumina Inc.) with 27 paired-end cycles to generate count files according to NanoString Next-Generation Sequencing (NGS) readout guidelines.

The Fastq files generated from NGS were converted into dcc files via Illumina BaseSpace GeoMx NGS Pipeline Version 2.0.0. GeoMx DSP Analysis Suite Version 2.2.0.111 was utilized for imputing the converted dcc files and performing data and statistical analysis. A confident detection threshold was set at geomean plus two standard deviations of negative probes. Technical signal and background quality checks were performed following NanoString recommendations. Gene counts were q3 normalized considering all target values. For DEG between melanoma and microglial cells, a linear mixed model (LMM) was implemented to account for ROI multiplicity with Benjamin–Hochberg corrections. A *p*-value less than 0.05 was considered statistically significant.

### 2.25. Biostatistic Analysis

Data were analyzed using Student’s *t*-test and considered significant at *p*-value ≤ 0.05. Bar graphs represent mean and standard error of the mean (SEM) across multiple independent experimental repeats.

## 3. Results 

### 3.1. A Transcriptomic Signature of Microglia Cells Reprogrammed by MBM 

**Figure 1:**
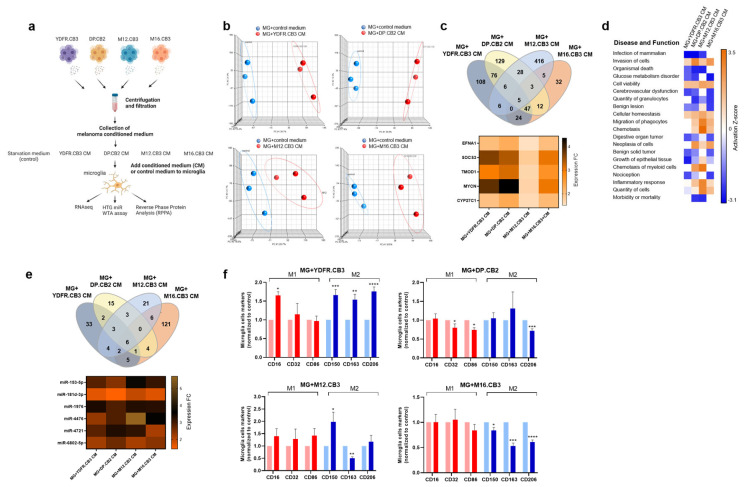
Melanoma-soluble factors regulate the molecular expression profile of microglia cells. (**a**) Scheme of the assays performed to characterize the molecular expression of microglia cells exposed to MCM of four melanoma brain metastatic cell lines. (**b**) Principal component analysis (PCA) of mRNA samples sequenced on an Illumina NextSeq 550. Each node signifies one biological replicate of control or MCM-treated microglia mRNA. Replicates of the same condition are clustered in the ellipsoids. (**c**) A Venn diagram illustrates an overlap of DEGs (*p*-value < 0.05, fold-change (FC) ≤ −1.5 or FC ≥ 1.5) in microglia cells treated with the four different MCM. Below, a heatmap of common DEGs. (**d**) Top disease pathways and biological functions enriched for genes significantly regulated in microglia following MCM treatment, as obtained by the Ingenuity Pathway Analysis (IPA) software, using comparison analysis. The net effects of gene expression changes on pathway activation or repression were determined using activation z-scores (threshold, z-score ≤ −2 or z-score ≥ 2). Color and intensity correspond to downregulated (blue) and upregulated (orange) pathways. (**e**) RNA samples of control or MCM-treated microglia cells were sequenced on an Illumina MiSeq platform. A Venn diagram illustrates an overlap of differentially expressed miRNA (*p*-value < 0.05, fold-change (FC) ≤ −1.25 or FC ≥ 1.25) in microglia cells treated with the four different MCM. Below, a heatmap of common differentially expressed miRNAs. (**f**) GFP-expressing microglia cells were co-cultured for 24 h with melanoma cells. The expression of three M1 markers (CD16, CD32, and CD86) and three M2 markers (CD150, CD163, and CD206) was tested using flow cytometry in the GFP-expressing microglia cells. The bars represent the average %positive cells (normalized) + SEM. Light bars: control microglia, dark bars: microglia co-cultured with melanoma cells. * *p* < 0.05, ** *p* < 0.01, *** *p* < 0.005, **** *p* < 0.001.

In order to characterize MBM-induced alterations in microglia, we profiled the transcriptome and proteome of microglia subjected to factors secreted from four different MBM cell lines (YDFR.CB3, DP.CB2, M12.CB3, and M16.CB3, see illustration in Figure 1a).

Total RNA extracted from microglia cells pre-treated with MCM of the four variants or with starvation medium (control) was sequenced on Illumina NextSeq 550. A principal component analysis (PCA) of the results revealed that the gene signature of microglia cells exposed to MCM of each of the four melanomas largely differed from that of the respective untreated microglia cells (Figure 1b). Moreover, each of the four melanomas induced a different signature in microglia. A total of 272, 306, 469, and 128 DEGs were found in microglia cells treated with MCM from YDFR.CB3, DP.CB2, M12.CB3, and M16.CB3 cells, compared to control microglia cells (*p*-value < 0.05 and FC ≤ −1.5 or FC ≥ 1.5). Comparing the four lists of DEG, we found that the expression of five genes: Ephrin-A1 (EFNA1), Suppressor of cytokine signaling 3 (SOCS3), Tropomodulin 1 (TMOD1), MYCN Proto-oncogene (MYCN), and Cytochrome P450 family 27 subfamily C member 1 (CYP27C1) was upregulated in microglia exposed to MCM from each of the four melanomas (Figure 1c).

Top disease pathways and biological functions (z-score ≤−2 or ≥2) associated with the significantly up- or downregulated genes in microglia treated with MCM were identified by ingenuity pathway analysis (IPA) (Figure 1d). This analysis indicated a uniform pattern of activation/inhibition of several biological functions in microglia treated with MCM of at least three of the four melanoma cell lines, including: invasion, migration (of phagocytes), viability, glucose metabolism, cellular homeostasis, chemotaxis, and inflammation. These altered pathways and molecules could have a profound impact on microglia–melanoma cross talk and, thereby, on the phenotype of the melanoma cells. 

### 3.2. Reprograming of Microglial miRNA Expression by Melanoma Impacts M1/M2 Polarization 

Post-transcriptional regulation of gene expression was mediated by miRNA. To test the involvement of miRNA in melanoma-mediated regulation of the microglia transcriptome, we compared the miRNA expression pattern of microglia treated with MCM from each of the four melanoma cell lines to that of control microglia cells. The HTG miR WTA assay was employed to profile 2083 miRNAs. 

A total of 56, 34, 45, and 145 miRNAs were differentially expressed in microglia cells treated correspondingly with MCM from YDFR.CB3, DP.CB2, M12.CB3, and M16.CB3 cells (Figure 1e), compared to untreated control microglia cells (*p*-value < 0.05 and FC ≤ −1.25 or FC ≥ 1.25). 

Six miRNAs (miR-153-3p, miR-181d-3p, miR-1976, miR-4476, miR-4721, and miR-6802-5p) were upregulated in microglia cells treated with MCM from all four melanoma cell lines. However, most alterations in miRNA expression were either unique to a single MBM cell line or restricted to some but not all of the cell lines. MCM from M12.CB3 and M16.CB3 melanomas upregulated miR-124-5p. Upregulated expression of this microRNA is associated with the M2 anti-inflammatory, cancer-promoting phenotype of microglia [37] and with a reduction in human M1 pro-inflammatory macrophage functions [38]. In cancer cells, this miRNA serves as a tumor suppressor [39]. These two opposing findings support the currently widely accepted notion that some factors may function either as tumor-promoting or tumor-restraining agents, depending on the context in which they are expressed [40]. 

Similarly, MCM from YDFR.CB3 and DP.CB2 melanomas upregulated the expression of miR-200b-3p in microglia. miR-200b-3p also manifests opposing functions with respect to tumor progression. While acting as a tumor suppressor when expressed by breast cancer cells [41], it supports tumor progression by promoting M2 polarization in cancer-associated macrophages [42]. 

By integration of RNA-seq and miRNA-seq data (Figure S1), we created putative interaction nets demonstrating the links between DEGs and their miRNA regulators in microglia cells exposed to CM of each melanoma cell line. In the DEG-miRNA interactomes established for both YDFR.CB3 and DP.CB2, miR-200b-3p is a central regulator of DEGs, as it regulates 41 out of 74 (55%) target genes in YDFR.CB3 CM-treated microglia, and 39 out of 52 (75%) target genes in DP.CB2 CM-treated microglia. In both interactomes, the miR-200b-3p node is connected to SOCS3. 

### 3.3. MBM Influences the M1/M2 Phenotype of Microglia

In view of these results, we tested the direct effects of co-culturing the four MBM cell lines with microglia on the expression of CD16, CD32, and CD86 (M1 biomarkers) and CD150, CD163, and CD206 (M2 biomarkers) on microglia cells (Figure 1f). Microglia cells co-cultured with YDFR.CB3 cells exhibited an upregulated expression of CD16, CD150, CD163, and CD206, compared to control microglia. Microglia cells co-cultured with DP.CB2 cells exhibited a downregulated expression of CD32, CD86, and CD206. Microglia cells co-cultured with M12.CB3 cells exhibited an upregulated expression of CD150 and a downregulated expression of CD163.These results indicate a mixed M1/M2 phenotype. Microglia cells co-cultured with M16.CB3 cells exhibited a downregulated expression of CD150, CD163, and CD206, indicating polarization towards the M1 phenotype. 

The spectrum of macrophage-activation signatures observed may be attributed to different and varying combinations of stimuli delivered by the different melanoma cell lines.

### 3.4. Melanoma-Derived Factors Control the Expression of SOCS3 in Microglia 

As mentioned above, SOCS3 was one of the five microglial genes that were upregulated by MCM from the four melanomas used in this study. In view of its role as an endogenous regulator of various signaling pathways including the Janus kinase (JAK)-signal transducer and activator of transcription (STAT) pathway [43] and as an immunosuppressor, we focused on the involvement of SOCS3, if any, in the microglia–melanoma crosstalk. 

**Figure 2:**
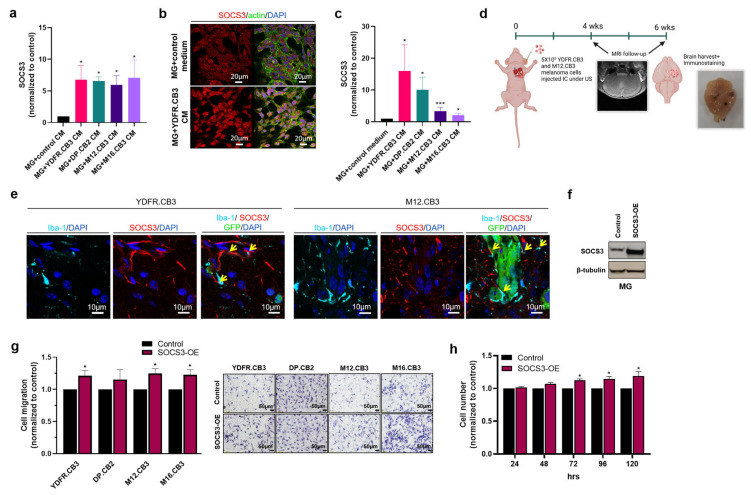
SOCS3 expression in microglia is upregulated by MCM and mediates tumor-associated properties in interacting melanoma cells. (**a**) The relative expression of SOCS3 mRNA in microglia cells incubated with MCM for 24 h was detected by RT-qPCR. RS9 was used for gene expression normalization. Data are presented as mean + SEM of biological replicates. * *p* < 0.05. (**b**) Representative images of microglia cells treated with YDFR.CB3 CM for 4 h stained with anti-SOCS3 Abs (red). Phalloidin-FITC was used for actin labeling (green). Magnification: ×20, scale bar: 20 µm. (**c**) The relative expression of SOCS3 mRNA in microglia^GFP^ cells isolated using fluorescence-activated cell sorting (FACS) following co-culture with melanoma^mCherry^ cells was detected by RT-qPCR. RS9 was used for gene expression normalization. Data are presented as mean + SEM of biological replicates. * *p* < 0.05, *** *p* < 0.005. (**d**) Brain metastasis in nude mice was generated by intracardiac (IC) inoculation of 5X105 human melanoma cells under a small animal ultrasound (US), and brain metastasis formation was followed-up by MRI. (**e**) Brain sections of a mice intracardially inoculated with YDFR.CB3^GFP^ or M12.CB3^GFP^ cells. Brain sections were stained by immunofluorescence for SOCS3 (red) and Iba-1 (cyan). Tumor cells (GFP) are in green. Cell nuclei were stained with DAPI (blue). Yellow arrows indicate co-expression of Iba-1 and SOCS3. Magnification: ×63, scale bar: 10 or 20 μm. (**f**) Western blot analysis of SOCS3 and β-tubulin in SOCS3 transduced microglia cells (SOCS3-OE) and control microglia cells. (**g**) Migration assay of melanoma cells towards SOCS3 overexpressing (SOCS3-OE) or control microglia cells. The migrated cells were fixed and counted in 6–10 fields. Data are presented as mean + SEM of biological replicates. * *p* < 0.05. Representative images are presented (10× magnification, scale bar: 50 μm). (**h**) YDFR.CB3 cells were co-cultured with SOCS3-overexpressing (SOCS3-OE) or control microglia cells in 96-well plates for 120 h. Cell number was determined using the IncuCyte system. * *p* < 0.05.

The upregulated expression of SOCS3 in microglia treated with MCM from the four melanoma cell lines was validated by RT-qPCR (Figure 2a). 

The use of confocal microscopy also validated the upregulated protein expression of SOCS3 in microglia treated for 4 h with MCM of all four melanoma cell lines (Figure 2b). 

We next asked if physical contact (by co-culturing) of microglia cells with the melanoma cells would also upregulate SOCS3 expression. Microglia^GFP^ cells were grown for 24 h with mCherry+ melanoma cells. Microglia^GFP^ cells were sorted using FACS and analyzed for SOCS3 expression. Figure 2c demonstrates that SOCS3 expression was significantly upregulated in microglia co-cultured with all four melanoma cells. We, thus, concluded that the transcriptomic signature of microglia following their interaction with melanoma cells may be conferred upon by melanoma-derived extracellular factors as well as by physical contact between these cells. 

### 3.5. SOCS3-Expressing Microglia Cells in Melanoma Brain Metastases Are Activated 

We next asked if the gene signature of MCM-treated microglia is expressed by microglia that infiltrates MBM in vivo. MBM xenografts were established in mouse brains by intra-cardiac inoculation of YDFR.CB3 and M12.CB3 cells (as illustrated in Figure 2d) as previously described [27]. 

Immunostaining of MBM generated by an intra-cardiac inoculation of YDFR.CB3^GFP^ or M12.CB3^GFP^ cells revealed that SOCS3 was expressed in brain stroma and at lower levels in tumor cells (Figure 2e). SOCS3 was co-localized to Iba-1 positive cells at the metastatic site.

### 3.6. The Involvement of SOCS3 in Melanoma-Microglia Cross Talk

SOCS3, a regulator of cytokine signaling, acts differently in different cell populations modulating intracellular signaling cascades, preventing excessive signaling and restoring homeostasis [44]. In pathological conditions such as cancer, SOCS3 either promotes or restrains disease progression [45]. In view of its important role in cancer biology, we asked if microglial SOCS3 is functionally involved in the melanoma-microglia crosstalk. 

To approach this question, we stably overexpressed SOCS3 in microglia cells (see Materials and Methods) (Figure 2f).

Migration assays, in which melanoma cells migrated through collagen-coated transwells towards control or SOCS3-overexpressing microglia, demonstrated that YDFR.CB3, M12.CB, and M16.CB3 melanoma cells migrated towards SOCS3 overexpressing microglia cells much more effectively than towards control microglia (Figure 2g). SOCS3 overexpression did not affect DP.CB2 migration.

To determine if SOCS3 expression by microglia affects melanoma cell proliferation, microglia^GFP^ cells overexpressing SOCS3 were co-cultured with the four melanoma^mCherry^ cell lines in the IncuCyte system and counted. The results show that SOCS3-overexpressing microglia supported the proliferation of YDFR.CB3 cells better than control microglia (Figure 2h). No effect on melanoma proliferation was observed in the other melanoma cell lines.

Taken together we tentatively conclude:

1. The four melanomas tested secrete factors that positively regulate the expression of microglial SOCS3.
2. This suppressor of cytokines does not play a major direct role in the cellular communication between melanomas and neighboring microglia. Nonetheless, microglial SOCS3 may play an indirect role in the progression of brain-metastasizing melanoma cells by its immunosuppressive functions [46].

### 3.7. Melanoma-Derived Factors Activate Microglial STAT3 

**Figure 3:**
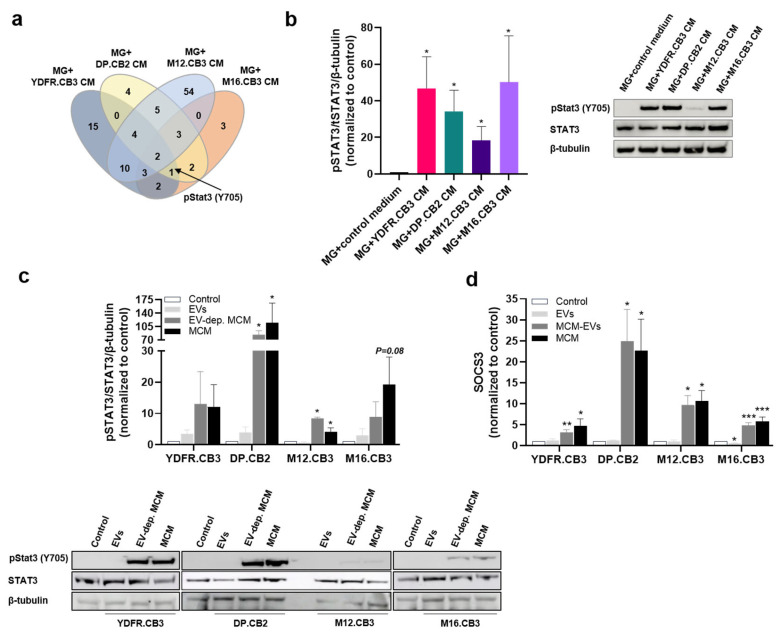
Melanoma-soluble factors regulate protein expression in microglia cells. (**a**) Protein lysates of control or MCM-treated microglia cells were analyzed for the expression of 295 proteins with RPPA. A Venn diagram illustrates an overlap of differentially expressed proteins (fold-change (FC) ≤ −1.2 or FC ≥ 1.2) in microglia cells treated with the four different MCM. (**b**) Western blot analysis of pSTAT3, STAT3, and β-tubulin in microglia cells incubated with MCM for 4 h. Data are presented as mean + SEM of biological replicates. * *p* < 0.05. (**c**) Western blot analysis of pSTAT3, STAT3, and β-tubulin in microglia cells incubated with EVs, EV-depleted MCM (EV-dep. MCM), or MCM of the 4 melanoma cell lines for 5 min. Data are presented as mean + SE.M of biological replicates. * *p* < 0.05. Note: the control sample in the middle blot (showing microglia treated with DP.CB2 and M12.CB3) is shared by microglia treated by both these cells. (**d**) The relative expression of SOCS3 mRNA in microglia cells treated with EVs, EV-depleted MCM, or MCM for 24 h was detected by RT-qPCR. RS9 was used as reference for gene expression normalization. Data are presented as mean + SEM of biological replicates. * *p* < 0.05, ** *p* < 0.01, *** *p* < 0.005.

Reverse phase protein array (RPPA) analysis [47] was performed in order to identify proteins involved in melanoma-mediated regulation of microglia cells. Compared to control microglia cells, a total of 37, 21, 81, and 16 proteins were differentially expressed in microglia cells treated correspondingly with MCM from YDFR.CB3, DP.CB2, M12.CB3, and M16.CB3 cells. For each pair of samples (MCM-treated vs. control microglia), we established a list of proteins that were differentially expressed by a FC ≤ −1.2 or FC ≥ 1.2. A Venn diagram (Figure 3a) demonstrated that the phosphorylation of Tyr705 in STAT3 was enhanced in microglia cells treated with MCM obtained from YDFR.CB3, DP.CB2, and M16.CB3.

The levels of phosphorylated STAT3 Tyr705 in microglia treated with MCM from M12.CB3 cells were lower than the cutoff point; however, the expression of total STAT3 was upregulated by 1.39-fold in these cells. Western blot analysis validated the RPPA findings: MCM from all four melanoma cell lines significantly upregulated STAT3 phosphorylation in microglia (Figure 3b).

STRING analysis confirmed that STAT3 is a central component in the interaction network between the up- or downregulated proteins (Figure S2).

Based on these results, and since microglial STAT3 is altered following interactions with several types of brain malignancies [48,49], we deduced that STAT3 is a pivotal factor in melanoma-microglia interactions.

### 3.8. Interleukin-6 (IL-6) in the Melanoma Secretome Activates STAT3 and Upregulates SOCS3 in Microglia

In order to identify the melanoma-derived factor(s) that induce STAT3 phosphorylation and SOCS3 upregulation, we fractionated the MCM from the four different melanoma cell lines to extracellular vesicles (EVs) and soluble fraction (SF or MCM depleted of EVs). The unfractionated MCM (containing both SF and EVs) served as starting material.

Microglia cells were treated with each of these fractions diluted in starvation medium for 5 min. The unfractionated MCM and the SF of DP.CB2 and M12.CB3 cells induced a significant STAT3 phosphorylation in microglia. Due to, possibly, large differences between the experimental repetitions, we did not observe a significant induction of STAT3 phosphorylation in microglia by unfractionated MCM and the SF derived from YDFR.CB3 and M16.CB3 cells. EVs extracted from the four MBMs did not induce STAT3 phosphorylation in microglia (Figure 3c). SOCS3 was also upregulated in microglia cells following treatment with unfractionated MBM or SF originating in all four melanoma cell lines but not by the EV fraction (Figure 3d).

**Figure 4:**
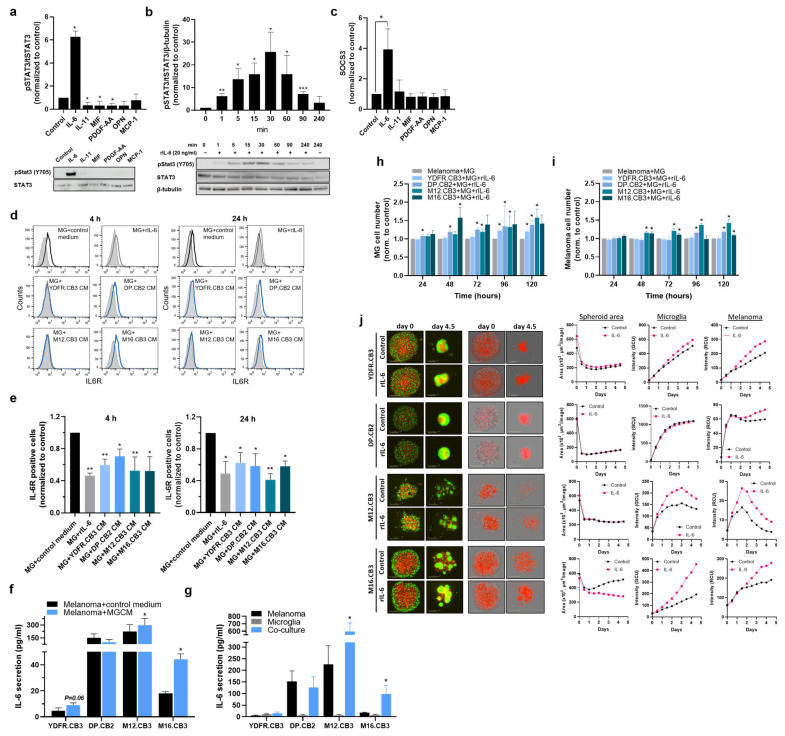
IL-6 is a regulator of melanoma-microglia interactions via the STAT3/SOCS3 pathway. (**a**) Western blot analysis of pSTAT3 and STAT3 in microglia cells incubated with rIL-6 (20 ng/mL), IL-11 (10 ng/mL), MIF (10 ng/mL), PDGF-AA (20 ng/mL), OPN (100 ng/mL) or MCP-1 (100 ng/mL) for 4 h. Data are presented as mean + SEM of biological replicates. * *p* < 0.05. (**b**) Western blot analysis of pSTAT3, STAT3 and β-tubulin in microglia cells incubated with rIL-6 for the indicated time points. Data are presented as mean + SEM of biological replicates. * *p* < 0.05, ** *p* < 0.01, *** *p* < 0.005. (**c**) The relative expression of SOCS3 mRNA in microglia cells treated with recombinant IL-6, IL-11, MIF, PDGF-AA, OPN or MCP-1 for 4 h was detected by RT-qPCR. RS9 was used for gene expression normalization. Data are presented as mean + SEM of biological replicates. * *p* < 0.05. (**d**,**e**) IL-6R expression was determined using flow cytometry of microglia cells treated with rIL-6 or with MCM of the 4 variants for 4 or 24 h. (**d**) Representative flow cytometry histograms of IL-6R expression in microglia are shown. (**e**) The bars represent the average IL-6R% positive cells in each treatment normalized to control cells + SEM of biological replicates. * *p* < 0.05, ** *p* < 0.01. (**f**,**g**) Extracellular levels of IL-6 were determined by ELISA. (**f**) Melanoma cells were treated for 4 h with MGCM, then starved for additional 24 h before collecting the supernatants. Untreated melanoma cells served as control. Data are presented as mean + SEM of biological replicates. * *p* < 0.05. (**g**) Mono-cultures of melanoma or microglia cells and co-cultures consisting of these two cell types (total of 1.5 × 10^6^ cells seeded at a ratio of 1:1) were grown in starvation medium for 28 h before collecting the supernatants. The bars represent mean IL-6 levels (pg/mL) in three independent experiments. Significance was evaluated using Student’s *t*-test for IL-6 levels in co-cultures, compared to mono-cultured cells. * *p* < 0.05. (**h**,**i**) Melanoma and microglia cells were co-cultured in 96-well plates. Cultures were treated with rIL-6 for 120 h. Microglia^GFP^ (**h**) or melanoma^mCherry^ (**i**) cell number was determined using the IncuCyte system. Data are presented as mean + SEM of biological replicates. * *p* < 0.05. (**j**) Spheroid formation of mCherry-labeled melanoma cells and GFP-labeled microglia cells (1:1) with or without rIL-6, imaged for 4.5 days using the IncuCyte system. Representative images of the wells at the beginning and end point of the experiment are presented. Area, GFP intensity (GCU), and mCherry intensity (RCU) are presented in the graphs. Experiments were performed at least three times, in 4–6 replicates.

We next set to identify melanoma-derived factors that may induce STAT3 phosphorylation and SOCS3 upregulation in microglia. Six STAT3-inducing cytokines (IL-6, IL-11, MIF, PDGF-AA, OPN, and MCP-1), all expressed by melanoma [50,51,52,53,54], were tested for their ability to induce these alterations in microglia cells. The Western blot analysis demonstrated that only rIL-6 induced STAT3 phosphorylation (Figure 4a) in microglia.

The induction of STAT3 phosphorylation occurred in a time-dependent manner peaking at 30 min following exposure of microglia to rIL-6 (Figure 4b). Additionally, out of the six cytokines, only rIL-6 upregulated SOCS3 expression in microglia (Figure 4c).

A flow cytometry analysis revealed that microglia cells express IL-6R. Stimulation of microglia cells with rIL-6 downregulated IL-6R expression following 4 and 24 h of stimulation due probably to receptor degradation and recycling [55]. Treating microglia cells with MCM originating in all four melanomas also downregulated membrane expression of the IL-6 receptor (Figure 4d,e).

Since we observed that both melanoma-derived soluble factors as well as recombinant IL-6 activate the STAT3/SOCS3 pathway in microglia, we asked if IL-6 is present in the [55] of the melanoma cells and whether microglia regulate the secretion of this cytokine from melanoma.

IL-6 levels were measured by ELISA in culture supernatants of naïve (control) melanoma cells, in supernatants of naïve (control) microglia cells, in supernatants of melanoma cells exposed to MGCM, and in supernatants of melanoma-microglia co-cultures. The results demonstrated that IL-6 was secreted from all four melanoma cell lines. Exposure of melanoma cells to MGCM resulted in upregulated levels of IL-6 secreted from M12.CB3 and M16.CB3 cells, compared to the basal levels of secreted IL-6 from naïve (control) melanoma cells (Figure 4f). Comparing IL-6 secretion levels in melanoma-microglia co-cultures with IL-6 secreted from melanoma and microglia mono-cultures demonstrated that the co-cultures of microglia with M12.CB3 and M16.CB3 melanoma cells secreted significantly higher amounts of IL-6 than the corresponding mono-cultures (Figure 4g). These results show that some but not all melanoma-microglia interactions promoted IL-6 production.

### 3.9. IL-6-Promotes Proliferation of Melanoma and Microglia Cells in Co-Cultures

We next asked if IL-6 influences the cellular cross talk between melanoma and microglia. We employed 2D co-cultures of mCherry-labeled melanoma cells (from our four cell lines) with GFP-labelled microglia cells and asked if IL-6 affects cell proliferation of either cellular partner or of both. The results show that rIL-6 promoted the proliferation of microglia cells in each of the four melanoma-microglia co-cultures in at least one time point (Figure 4h). The response of microglia cells to IL-6 in terms of proliferation was the fastest in microglia grown with DP.CB2 cells, and the most delayed and the least effective in microglia cells grown with YDFR.CB3 cells. The measurement of melanoma cell number in the co-cultures demonstrated that rIL-6 enhanced the proliferation of DP.CB2, M12.CB3, and M16.CB3 cells, but not that of YDFR.CB3 cells (Figure 4i). The response of melanoma cells to IL-6 in the co-culture was the fastest in M12.CB3 and M16.CB3 cells. Taken together, rIL-6 impacts the proliferation of microglia in their co-culture with all melanoma cell lines; however, its impact on the proliferation of the melanoma cells in these co-cultures in heterogeneous.

To study the effect of IL-6 on melanoma-microglia interactions, we employed the 3D co-culture tumor spheroid model. Such models partially mimic the metastatic microenvironment of the brain [56].

mCherry-labeled cells from the four melanoma cell lines were co-cultured with GFP-labeled microglia cells to form spheroid cultures. Four-and-a-half days after seeding, we examined the impact of rIL-6 on the following parameters: spheroid area, GFP intensity (reflecting the quantity of microglia cells), and mCherrry intensity (reflecting the quantity of melanoma cells). The spheroid area was not affected in the 3D co-cultures, of microglia with YDFR.CB3, M12.CB3, and DP.CB2 melanoma cells. The microglia-M16.CB3 structures, which although not being able to form a round, full spheroid, were more compressed under IL-6 treatment (Figure 4j). The treatment of microglia-YDFR.CB3, microglia-M12.CB3, or microglia-M16.CB3 spheroids with rIL-6 resulted in an increase in the quantity of microglia cells. Enhanced microglia proliferation was not evident in the DP.CB2 containing spheroids. rIL-6 increased the quantity of melanoma cells in all four microglia–melanoma spheroids.

### 3.10. IL-6/JAK/STAT3 Pathway Inhibitors Restrain the Melanoma-Mediated Activation of Microglial STAT3 and SOCS3 Upregulation and Inhibit Proliferation of Both Melanoma and Microglia in Co-Cultures

The experiments reported above indicated that IL-6 is the functional microglia-activator in MCM and that this cytokine activates the microglial JAK/STAT3 pathway. In order to further validate these results, we employed the IL-6/JAK/STAT3 pathway inhibitors αIL-6R, Baricitinib (JAK inhibitor, Amadis Chemical) and Stattic (STAT3 Inhibitor, Sigma-Aldrich), and determined their effect on the melanoma-microglia crosstalk.

Western blot analysis demonstrated a heterogeneous response to the inhibition of IL-6-mediated activation of microglia. αIL-6R (which also binds cell-free IL-6R that may interact with IL-6 and also induce JAK/STAT3 signaling [57,58]) inhibits STAT3 phosphorylation in microglia cells treated with CM of M12.CB3 and M16.CB3 cells but not in microglia treated with CM of YDFR.CB3 and DP.CB2 cells. Since YDFR.CB3 cells secrete relatively low levels of IL-6 (Figure 4f,g), it is to be expected that the inhibition of STAT3 phosphorylation via IL-6R would be minimal.

**Figure 5:**
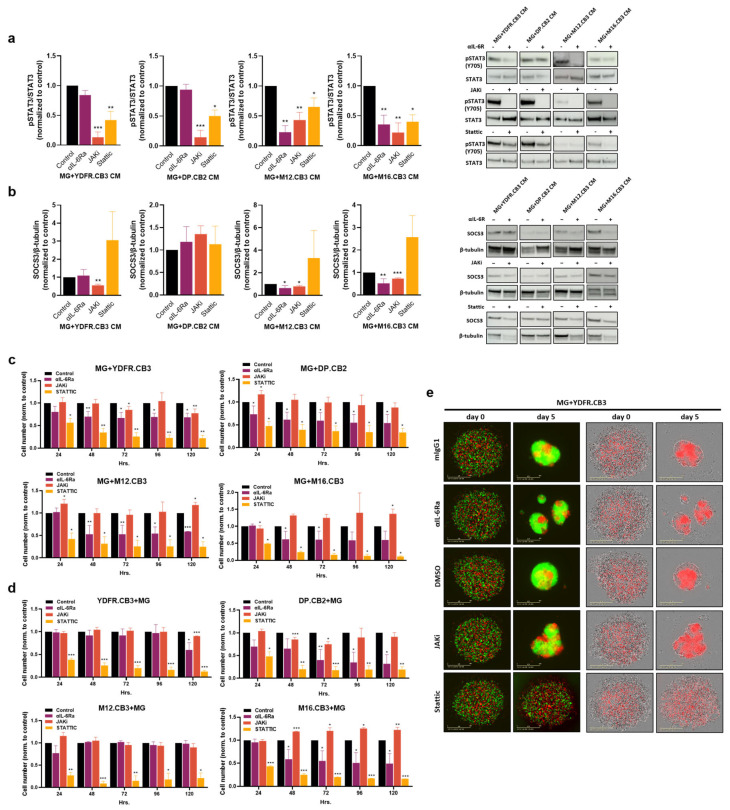
STAT3 inhibition in microglia modulates microglia–melanoma interactions. (**a**) Western blot analysis of pSTAT3 and STAT3 in microglia cells incubated with MCM and with either αIL-6R (or mouse IgG1 as control), Baricitinib (JAKi) or Stattic (or DMSO as control) for 4 h. Data are presented as mean + SEM of biological replicates. * *p* < 0.05, ** *p* < 0.01, *** *p* < 0.005. (**b**) Western blot analysis of SOCS3 in microglia cells incubated with MCM and with either αIL-6R, Baricitinib (JAKi) or Stattic for 4 h. β-tubulin was used for protein expression normalization. Data are presented as mean + SEM of biological replicates. * *p* < 0.05, *** *p* < 0.005. (**c**,**d**) Microglia^GFP^ and melanoma^mCherry^ were co-cultured and treated with αIL-6R, Baricitinib (JAKi) or Stattic. Microglia^GFP^ (**c**) or melanoma^mCherry^ (**d**) cell number was determined using the IncuCyte system. Data are presented as mean + SEM of biological replicates. * *p* < 0.05, ** *p* < 0.01, *** *p* < 0.005. (**e**) Spheroid formation of mCherry-labeled melanoma cells and GFP-labeled microglia cells (1:1) with αIL-6R, Baricitinib (JAKi), or Stattic (and hIgG1 or DMSO as controls), imaged for 5 days using the IncuCyte system. Representative images of the wells at the beginning and end point of the experiment are presented. Experiments were performed at least three times, in 4–6 replicates.

Baricitinib and Stattic were found to inhibit STAT3 phosphorylation in microglia cells treated with MCM derived from all four cell lines (Figure 5a).

To test whether this inhibition impacts SOCS3 expression by microglia, we treated microglia cells with MCM of the four melanomas together with the three inhibitors. αIL-6R reduced SOCS3 expression in M12.CB3 and M16.CB3 cells, similar to its effect on pSTAT3 in these cells. Baricitinib reduced SOCS3 expression in YDFR.CB3, M12.CB3, and M16.CB3 cells. Stattic did not affect SOCS3 expression in any of the cells (Figure 5b). These results indicate that, at least in some cases, the IL-6/JAK/STAT3 pathway is upstream to SOCS3 regulation.

We next measured the effect of the IL-6/JAK/STAT3 inhibitors αIL-6R, Baricitinib, and Stattic on the proliferation of melanoma and microglia cells in 2D and 3D co-cultures.

2D proliferation assay. Co-cultures of mCherry-labeled melanoma cells and GFP-labeled microglia cells were treated with the three inhibitors and cell number was determined. αIL-6R and Stattic inhibited the proliferation of microglia in the four co-cultures, while Baricitinib had an opposing, enhancing effect on microglia proliferation in co-cultures containing DP.CB2, M12.CB3, and M16.CB3 cells. Baricitinib inhibited microglia proliferation in YDFR.CB3 containing co-cultures (Figure 5c). Although αIL-6R did not show an inhibitory effect on STAT3 phosphorylation in microglia exposed to YDFR.CB3 CM, it did show an inhibitory effect on microglia proliferation when co-cultured with YDFR.CB3. This might be explained by the upregulated IL-6 levels observed in such co-cultures compared to IL-6 levels secreted by YDFR.CB3 alone (Figure 4g).

Examining melanoma cell number in the co-cultures showed that αIL-6R inhibited the proliferation of YDFR.CB3, DP.CB2, and M16.CB3 cell lines, while it had no effect on M12.CB3 proliferation. The inhibition of YDFR.CB3 proliferation with αIL-6R and Baricitinib was only observed after 120 h in co-culture. This may indicate that only after a prolonged period of time, an effective amount of IL-6 accumulated in the culture. Baricitinib inhibited the proliferation of YDFR.CB3 and DP.CB2 cells. Stattic inhibited the proliferation of all four melanoma cell lines (Figure 5d).

Spheroid 3D assay. We next tested the effect of IL-6/JAK/STAT3 pathway inhibition on the ability of YDFR.CB3 and DP.CB2 melanoma cells to form spheroids with microglia cells. An examination of the 3D melanoma-microglia structures was carried out five days after seeding. In the YDFR.CB3–microglia combination, treatment with αIL-6R resulted in the formation of fragmented spheroids, instead of a round organized structure as obtained in the control (Figure 5e). αIL-6R had no effect on DP.CB2-microglia spheroids. Baricitinib treatment reduced the eccentricity of YDFR.CB3–microglia spheroids, but had no effect on DP.CB2–microglia spheroids. Treatment with Stattic resulted in the most substantial alteration in 3D structures, as it completely abolished the ability of both melanomas to form spheroids.

The above results demonstrate that melanoma-mediated activation of the microglial IL-6/JAK/STAT3 pathway is a pivotal factor in microglial support in melanoma malignancy.

### 3.11. The JAK/STAT Signaling Pathway in Microglia in Melanoma Patients

**Figure 6:**
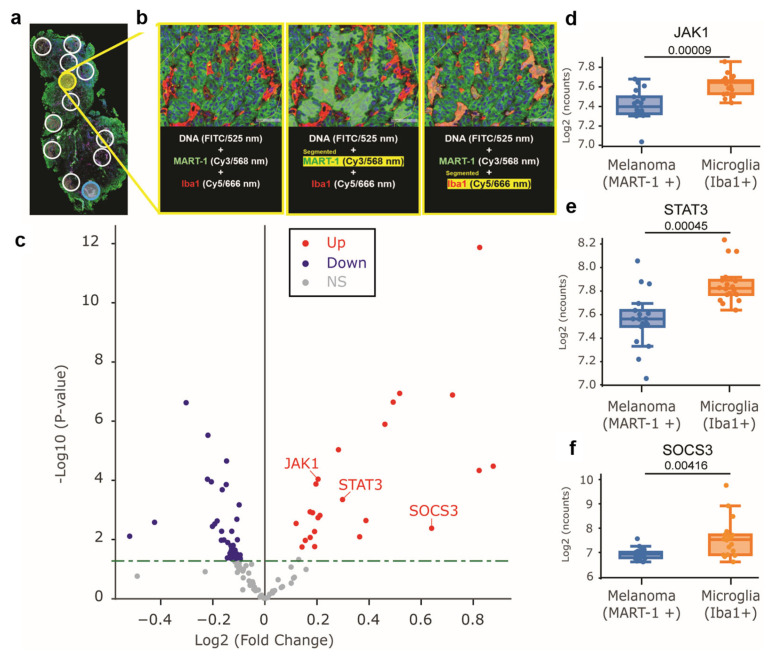
Transcriptomic analysis of MBM using NanoString GeoMx DSP. (**a**,**b**) Three MBM FFPE tissues were stained using fluorescent conjugated antibodies for MART-1 (Cy3, melanoma cells), Iba-1 (Cy5, microglia cells), and the DNA dyes SYTO13 (FITC, DNA). All tissues were processed with the NanoString GeoMx CTA probes to profile the transcriptome of over 1800 genes. Six regions of interest (ROI) were selected per tissue, masks were applied for MART-1 and Iba-1 segments. All the segments were UV-illuminated to release oligos. A total of 18 ROIs were collected for MART-1- or Iba-1 positive segments. (**c**) Volcano plot showing the expression of the 118 genes that are part of JAK/STAT signaling pathways in melanoma and microglia cells from MBM patients. Upregulated genes (Up) are shown in red, downregulated genes (Down) are shown in blue, and genes that did not significantly change (NS) are shown in grey. (**d**–**f**) Boxplot showing the mRNA expression (Log2 normalized counts (n counts)) of JAK1 (**d**), STAT3 (**e**), and SOCS3 (**f**). NS: not significant.

To determine the transcriptomic profiles of microglia cells, three human FFPE tissues from patients diagnosed with MBM were selected. The tissues were analyzed using NanoString GeoMx Digital Spatial Profiler (DSP) with the CTA probes for the transcriptomic profiling of over 1800 genes. This assay provided spatial transcriptomic analysis that relies on cell identification by specific morphological markers. Briefly, the morphological markers selected were MART-1 (Cy3, melanoma cells), Iba-1 (Cy5, microglia cells), and SYTO13 (FITC, DNA). Six regions of interest (ROI) were selected for MART-1- or Iba-1-positive segments per patient (Figure 6a,b). Masks were applied and only MART-1- or Iba-1-segmented areas were UV-light illuminated for releasing oligos. A total of 18 segments were collected for MART-1 or Iba-1, and they were then sequenced.

The transcriptomic profiles of melanoma and microglia cells were compared to determine differences in the JAK1/STAT3 signaling pathway. To achieve that, we focused on the 118 genes characterized by the CTA probes that are part of the JAK1/STAT3 signaling pathway. Microglia cells showed significantly higher mRNA expression for JAK1, STAT3, and SOCS3 compared to melanoma cells (Figure 6c–f). These results confirm our previous in vitro and in vivo observations of a significant role for the JAK/STAT pathway activation in microglia cells of MBM.

## 4. Discussion

It is well accepted that “dynamic reciprocity” in the crosstalk between tumors and their microenvironment includes not only the ECM-tumor interactions as originally envisaged by Bissell et al. [59] but all cellular and molecular components of the tumor microenvironment (TME). These include resident and infiltrating cells such as fibroblasts, lymphocytes, myeloid cells, the secretome of all these cells such as cytokines and chemokines [60], as well as components residing in distant sites, e.g., the endocrine system [61].

The continuous, dynamic, evolving, and bidirectional interactions impact both the cancer cells as well as the non-cancerous cells in the TME. These cells respond to the interactive signaling by reprograming their molecular and functional phenotype. This continuous cycle is a major determinant of tumor progression towards metastasis [4,60].

This reality boosted efforts to identify novel biomarkers and targets for diagnostic and therapy purposes both on cancer cells as well as on non-cancerous microenvironmental components [62,63,64]. Indeed, the targeting of non-cancerous TME components became the focal point of numerous therapy trials due to the clinical successes of the immune checkpoint inhibition blockade [65] and CAR T-cell therapy [66].

On their way to colonize the brain parenchyma, brain-metastasizing melanoma cells encounter constituents of the unique brain microenvironment such as cells of the blood–brain barrier, astrocytes, microglia, and neurons.

In an effort to identify determinants of MBM, our lab studies explored the interactions between brain metastasizing melanoma cells and cells of the brain microenvironment [6,7,8,9,10,11,12,13,14,22,27,28,67]. Such interactive components could serve as theranostic biomarkers for MBM.

Microglia, the resident macrophages of the brain, are the main immunological component in the CNS. These cells mediate inflammatory and immunological responses in a variety of pathological conditions such as stroke, neurodegenerative diseases, and CNS malignancies [68,69]. A histological examination of clinical human MBM and human MBM xenografts revealed a substantial infiltration of microglia into these lesions. Melanoma also reprograms the transcriptome of microglia. These results led us to hypothesize that microglia–melanoma interactions are central for the development of MBM [8].

The aim of the present study was to decipher the molecular pathways that generate melanoma-triggered reprograming of the microglia transcriptome.

In view of previous reports indicating an inter-melanoma heterogeneity with respect to functional responses of melanoma cells to various stimuli [6,12,27], we recognized the need to employ multiple cell lines in studies such as the present one. Four different melanoma cell lines were, therefore, employed in the present study.

An extensive characterization of the impact of melanoma cells and their secretome on the molecular signature of microglia cells revealed that melanoma-microglia reciprocal interactions activate STAT3 signaling in microglia cells, in a pattern unique to each MBM cell line, thereby sketching an individual malignant phenotype of each of the four interacting melanomas.

Soluble factors secreted by brain-colonizing melanoma cells induce a vicious cycle in which both melanoma as well as microglia cells respond to signals delivered by the interacting partner resulting in a mutual phenotype remodeling and reprograming.

Specifically, factors secreted by four MBM cell lines induced STAT3 phosphorylation in the Tyr705 residue in microglia. Notably, the induction was more prominent following exposure to soluble factors from three melanomas, and it was less prominent following exposure to a fourth melanoma (M12.CB3). Factors secreted by four MBM cell lines uniformly altered the expression of five genes in microglia including the STAT3 target gene SOCS3 [70].

STAT3 was initially identified in hepatocytes as acute-phase response factor (APRF). When activated by IL-6, it regulates inflammatory responses via binding to promoters of acute-phase genes. STAT3 is activated in cancer cells as well as in non-cancerous cells. Its activation in cancer cells, including melanoma, as well as in microenvironmental cells, promotes growth rate and migratory ability of the cancer cells and enhances metastatic spread [71,72]. Its inhibition attenuates cancer progression [73].

STAT3 signaling pathway is suppressed in M1-type macrophages but activated in M2-type macrophages and microglia [74,75]. Our findings indicated that miR-124-5p is upregulated in microglia by factors secreted from two melanomas (M12.CB3 and M16.CB3) and miR-200b is upregulated in microglia by factors secreted from the two other melanomas (YDFR.CB3 and DP.CB2). miR-124-5p and miR-200b-3p may, therefore, drive tumor progression by inducing macrophage polarization to the M2 phenotype. M2 microglia promotes progression of brain cancer by increasing proliferation and survival of the cancer cells and by inducing anti-inflammatory, pro-tumorigenic responses [76].

Based on the regulatory functions of these miRNAs, we predicted that factors secreted from our four-melanoma cell lines would induce an M1 to M2 transition of microglia. However, the results of experiments measuring the effect of MCM on microglial M1/M2 markers demonstrated a heterogeneous response of microglia to the signals delivered by the four melanomas manifested by four different microglial M1/M2 phenotypes. Cytokines such as IL-4, IL-13, TGFβ, and IL-10 were most probably present in the melanoma-microglia interface and could have provided a variety of signals that led to divergent M1/M2 phenotypes [77]. Moreover, we also demonstrated that microglia may adopt intermediate M1/M2 phenotypes expressing both M1 and M2 markers confirming previous studies [78,79].

The four melanoma cell lines used in the present study secrete IL-6. In view of the fact that IL-6 influences the morphology, gene expression, and secretome of microglia [80,81,82], we hypothesized that melanoma-derived IL-6 binds to its receptor expressed by microglia cells to initiate a signaling cascade through the IL-6/STAT3 pathway.

In conformity with the results demonstrating that IL-6 modulates the response of microglia to a variety of signals [83], we found that individual MBM cell lines differ in IL-6 secretion level and in the regulation of its secretion. IL-6 also influenced differentially the interaction of microglia with the different melanomas. IL-6 addition to three melanoma-microglia co-cultures resulted in enhanced proliferation of both melanomas as well as microglia cells. In the YDFR.CB3-microglia co-culture, we observed a delayed and less prominent enhancement of microglia proliferation and no effect on melanoma proliferation. The influence of IL-6 on the proliferation of melanoma and microglia cells in 3D co-cultures was also heterogeneous. The proliferation of co-cultured microglia and DP.CB2 cells was not affected by IL-6. This suggests that IL-6 activates different pathways in melanoma 2D and 3D co-cultures.

SOCS3 is an important regulator of cancer progression through a variety of signaling pathways [84]. Depending on the type of cancer, SOCS3 may function as a tumor suppressor or as a promoter. Its inhibition leads to ovarian or breast cancer proliferation, invasion, and chemotherapy resistance. In murine melanoma, SOCS3 functions as a tumor suppressor [85]. In other types of cancer such as pancreatic cancer, prostate cancer, or glioblastoma, SOCS3 promotes tumor progression as its upregulation leads to tumor cell proliferation, migration and angiogenesis [84]. In murine melanoma, SOCS3 functions as a tumor suppressor [85].

The information of the functions of SOCS3 in microglia is rather limited. It is known, however, that SOCS3 may serve as an endogenous attenuator of macrophage/microglial activation and gene expression [46].

In the present study, we demonstrated that brain-metastasizing tumor cells uniformly impact the expression of microglial SOCS3. However, microglial SOCS3, in turn, enhanced the migration and/or proliferation of three MBM cells out of the four cell lines used in this study. The migration and proliferation rate of DP.CB2 cells were not altered by SOCS3 over-expression in microglia.

The significance of STAT3 activation in the melanoma-microglia crosstalk was highlighted by findings that the IL-6/JAK/STAT3 pathway inhibitors restrained the melanoma-mediated activation of microglial STAT3 as well as the up-regulation of SOCS3 in microglia cells exposed to melanoma soluble factors. However, this inhibition was cell specific. The αIL-6R inhibitor neither inhibited STAT3 phosphorylation nor SOCS3 up-regulation in YDFR.CB3 and DP.CB2 cells. Moreover, these inhibitors affected the proliferation of microglia and melanoma cells differently. The three inhibitors decreased the proliferation of microglia cells co-cultured with YDFR.CB3 melanoma cells. In the other three co-cultures, two inhibitors, αIL-6R and Stattic, also decreased the proliferation of microglia; however, Baricitinib enhanced the proliferation of microglia. We also observed heterogeneity with respect to the effect of inhibitors on melanoma proliferation. Taken together, these results show that SOCS3 was involved in the progression of some but not all the four melanomas employed in this study.

**Figure 7:**
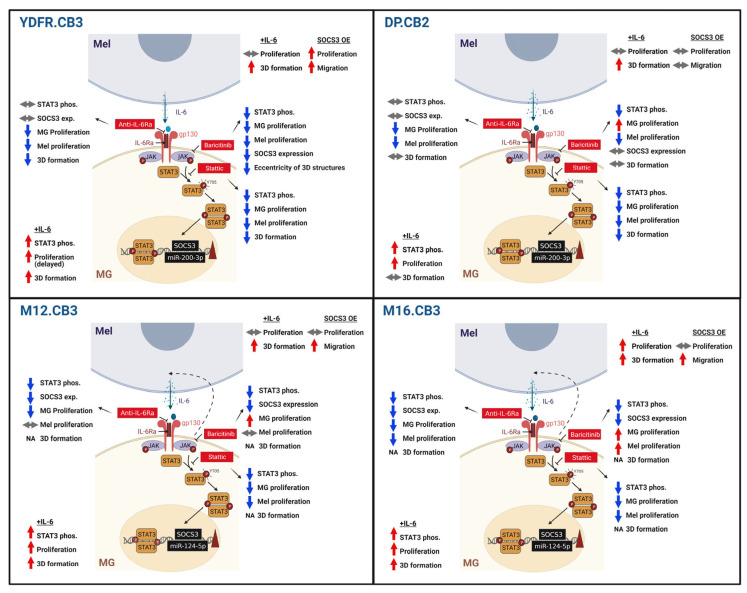
Summary of the role of the IL-6/STAT3 pathway in the crosstalk between microglia and the four different MBM cell lines. The reciprocal effects in the microglia–melanoma crosstalk, as obtained by integrating the study results, demonstrating a reprogramming of microglia by melanoma via different variations of the IL-6-mediated activation of STAT3. These, in turn, promote the malignant phenotype of melanoma cells by a vicious signaling cycle. OE: overexpression, NA: not available.

The integration of the data presented in this study show that melanoma cells prompt microglia reprogramming via different variations of IL-6-mediated activation of STAT3 (Figure 7). This activation, in turn, promotes the malignant phenotype of melanoma cells by a vicious signaling cycle.

The heterogeneous response of non-cancerous microenvironmental cells (such as microglia) to cancer-derived cues poses a challenge to pre-clinical and clinical strategies aiming to target cancer-promoting functions mediated by TME components. Individualized anti-metastatic therapy may have to be tailored based on results of pre-treatment in vitro assays analyzing the functional consequences of interactions between cancer and non-cancerous cells in the shared microenvironment.

Inter-tumor heterogeneity due to genomic and epigenetic differences between cancer patients [86,87,88] is manifested by disparities in various features of the tumors such as gene expression [89] or response to external signals [6,12].

In this study, inter-tumor heterogeneity is manifested by distinct influences of four melanoma cell lines on human microglia cells. While all four melanoma cell lines activated a number of molecular pathways in microglia, other pathways were activated by only some melanoma cell lines. It is, therefore, not unlikely that multiple pathways may drive melanoma towards brain metastasis [22].

Translated to the clinical level, the results of the present study accentuate the need for individualized precision theranostics with respect to MBM.

## Data Availability

Data were submitted to the GEO database. Accession numbers will be provided when available.

## Supplementary Materials

The following supporting information can be downloaded at: https://www.mdpi.com/article/10.3390/cells12111513/s1, Supplementary Figure S1: Interactome demonstrating the relationships between differentially expressed miRNAs and DEGs in microglia treated with MCM; Supplementary Figure S2: STRING analysis of differentially expressed proteins in microglia treated with MCM; Supplementary Table S1: List of antibodies utilized in the study; Supplementary Table S2: List of Oligonucleotide Primers used for RT-qPCR; Supplementary Table S3: List of antibodies utilized in NanoString GeoMx DSP.
