# Supplementary material for: The Vicious Cycle of Melanoma-Microglia Crosstalk: Inter-Melanoma Variations in the Brain-Metastasis-Promoting IL-6/JAK/STAT3 Signaling Pathway
Source: Cells. 2023 May 30;12(11):1513. doi: 10.3390/cells12111513 (PMC10253015; doi:10.3390/cells12111513)
Supplement: Supplementary file 1 [file cells-12-01513-s001.zip › Table S1 6.3.23.pdf]

Supplementary Table S1. List of antibodies utilized in the study.

Antibody	Catalogue no.	Origin[2]	Application	Concentration	Manufacturer details
Polyclonal Anti-AIF-1/Iba1	NB100-1028	Goat	IHC (frozen)	1:100	Novus Biologicals, Centennial, CO, USA
Polyclonal Anti-Alix	ab76608	Rb	WB	1:500	Abcam, Cambridge, MA, USA
Polyclonal Anti-beta Tubulin	ab6046	Rb	WB	1:1000	Abcam, Cambridge, UK
CD16 Antibody, anti-human, REAfinity™	130-113-951	Ms	FACS	1:50	Miltenyi Biotec, Bergisch Gladbach, Germany
CD32 Antibody, anti-human	130-127-008	Ms	FACS	1:50	Miltenyi Biotec, Bergisch Gladbach, Germany
Monoclonal Anti-CD63 (H5C6)	556019	Ms	WB	1:1000	BD Pharmingen, San Jose, CA, USA
Monoclonal Anti-CD81 (JS-81)	555675	Ms	WB	1:200	BD Pharmingen, San Jose, CA, USA
CD86 Antibody, anti-human, REAfinity™	130-116-264	Ms	FACS	1:50	Miltenyi Biotec, Bergisch Gladbach, Germany
CD150 (SLAM) Antibody, anti-human, REAfinity™	130-099-722	Ms	FACS	1:50	Miltenyi Biotec, Bergisch Gladbach, Germany
CD163 Antibody, anti-human, REAfinity™	130-112-287	Ms	FACS	1:50	Miltenyi Biotec, Bergisch Gladbach, Germany
CD206 Antibody, anti-human	130-124-012	Ms	FACS	1:50	Miltenyi Biotec, Bergisch Gladbach, Germany
Monoclonal Anti-Human IL-6R alpha	MAB227	Ms	FACS	1 µg/ml	R&D Systems, Minneapolis, MA, USA
Monoclonal Anti-Human IL-6R alpha	MAB227	Ms	Inhibition assay	1 µg/ml	R&D Systems, Minneapolis, MA, USA
Polyclonal Anti-SOCS3	ab16030	Rb	IHC (frozen)	1:50	Abcam, Cambridge, MA, USA
Polyclonal Anti-SOCS3	ab16030	Rb	WB	1:1000	Abcam, Cambridge, MA, USA
Polyclonal Anti-SOCS3	ab16030	Rb	IF	1:240	Abcam, Cambridge, MA, USA
Monoclonal Anti-phospho-Stat3 (Tyr705) (D3A7)	9145	Rb	WB	1:1000	Cell Signaling Technology, Inc., Danvers, MA, USA
Monoclonal Anti-Stat3 (124H6)	9139	Ms	WB	1:500	Cell Signaling Technology, Inc., Danvers, MA, USA

[2] Ms: mouse, Rb: rabbit.
