# Supplementary material for: The Vicious Cycle of Melanoma-Microglia Crosstalk: Inter-Melanoma Variations in the Brain-Metastasis-Promoting IL-6/JAK/STAT3 Signaling Pathway
Source: Cells. 2023 May 30;12(11):1513. doi: 10.3390/cells12111513 (PMC10253015; doi:10.3390/cells12111513)
Supplement: Supplementary file 1 [file cells-12-01513-s001.zip › Table S2.pdf]

Supplemental Table S2. List of Oligonucleotide Primers used for RT-qPCR.

Gene Symbol and Accession No.[1]	Sense Primer[2]	Anti-Sense Primer
RS9 (NM_001013)	5′-CGGAGACCCTTCGAGAAATCT-3′	5′-GCCCATACTCGCCGATCA-3′
SOCS3 (NM_003955)	5′-CCATTCGGGAGTTCCTGGAC-3′	5′-TTGGCTTCTTGTGCTTGTGC-3′

[1] RS9: Ribosomal subunit 9; SOCS3: Suppressor of cytsokine signaling 3.

[2] RT-qPCR primer sequences as obtained from the GenBank Nucleotide Database of the NCBI database.
