# Supplementary material for: The Vicious Cycle of Melanoma-Microglia Crosstalk: Inter-Melanoma Variations in the Brain-Metastasis-Promoting IL-6/JAK/STAT3 Signaling Pathway
Source: Cells. 2023 May 30;12(11):1513. doi: 10.3390/cells12111513 (PMC10253015; doi:10.3390/cells12111513)
Supplement: Supplementary file 1 [file cells-12-01513-s001.zip › Table S3.pdf]

Supplementary Table S3. List of antibodies utilized in Nanostring GeoMx DSP.

Antibody/Reagents	Catalogue no.	Wavelength (nm)	Concentration	Source
Melan-A/MART-1 (A103)	NBF2-46603AF532	532 (Cy3)	1:150	Novus Biologicals, CO, USA
CD45	FAB1430T-100	594 (Texas Red)	1:100	R&D Systems, MN, USA
Iba1	MABN92-AF647	647 (Cy5)	1:100	EMD Millipore, CA, USA
SYTO 13	S7575	488 (FITC)	1:2000	ThermoFisher, MA,USA
