# Supplementary figures and images for: The Vicious Cycle of Melanoma-Microglia Crosstalk: Inter-Melanoma Variations in the Brain-Metastasis-Promoting IL-6/JAK/STAT3 Signaling Pathway
Source: Cells. 2023 May 30;12(11):1513. doi: 10.3390/cells12111513 (PMC10253015; doi:10.3390/cells12111513)

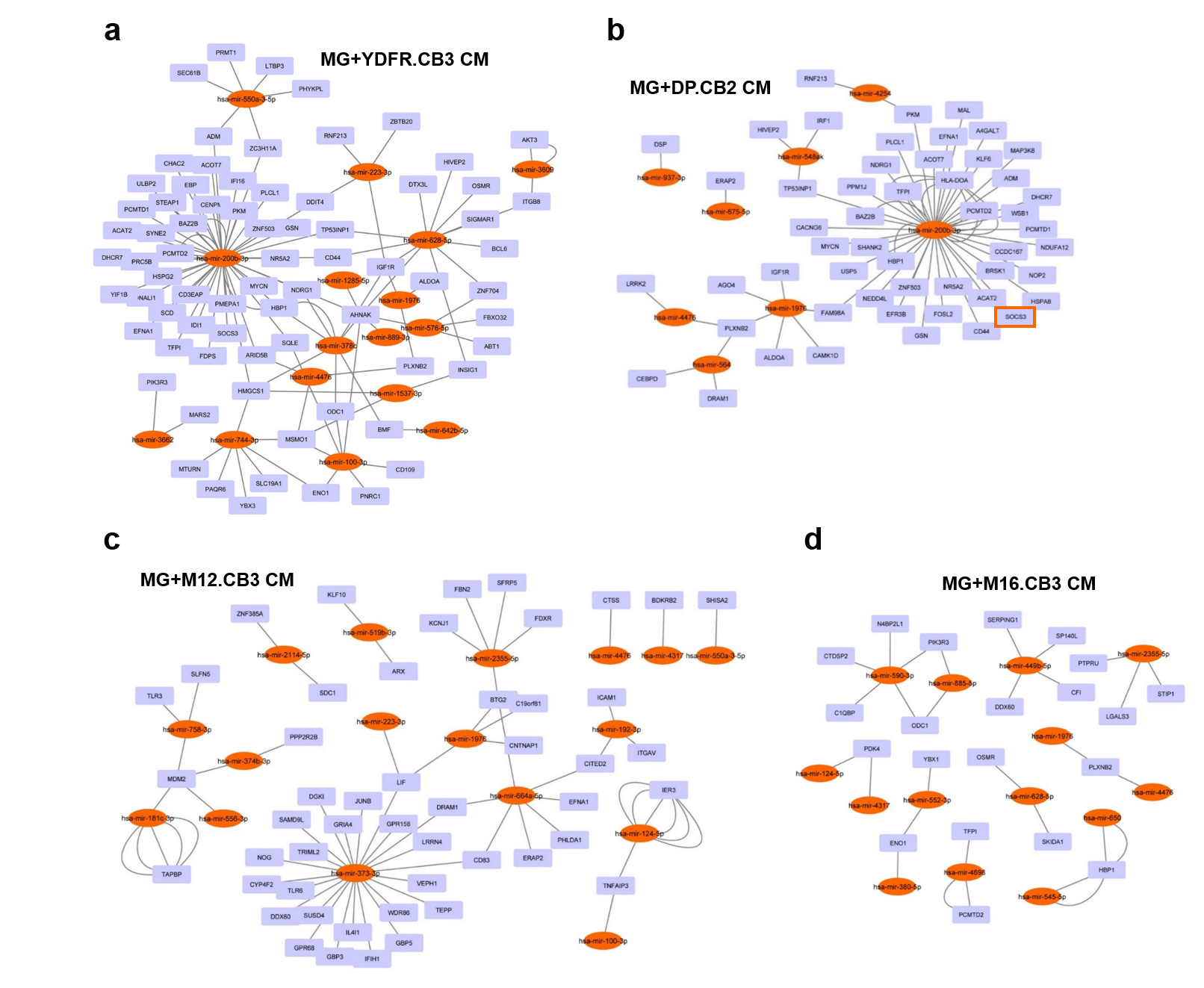

Supplement: Supplementary file 1 [file cells-12-01513-s001.zip › Supplementary Figure S1.tif]

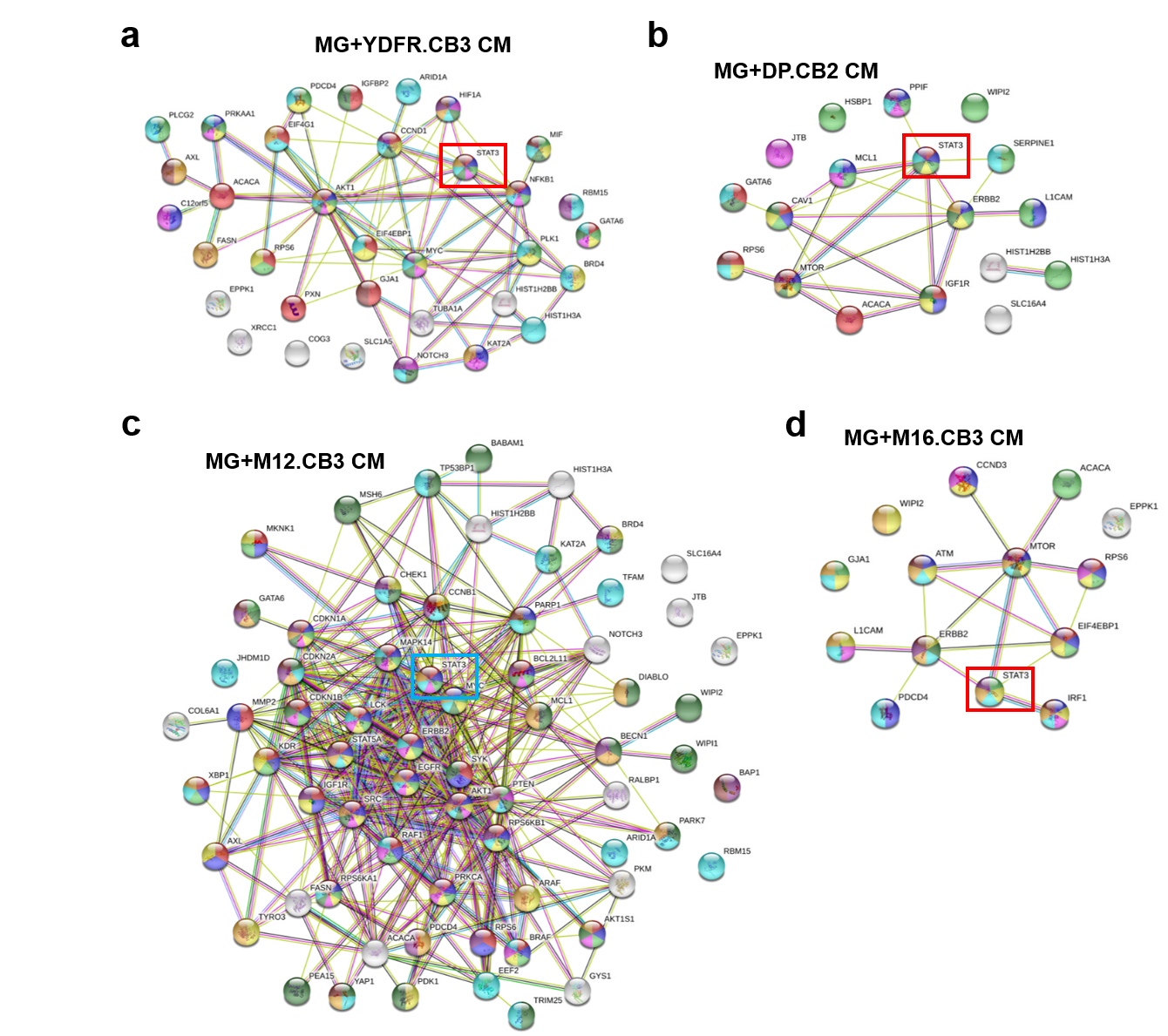

Supplement: Supplementary file 1 [file cells-12-01513-s001.zip › Supplementary Figure S2.tif]
